# Fournier’s Gangrene Caused by Actinomyces europaeus in a Young Male With Poorly Controlled Diabetes Mellitus: A Case Report

**DOI:** 10.7759/cureus.103379

**Published:** 2026-02-10

**Authors:** Mohammed S Alam, Mahfuza Khan, Roxana Lazarescu, Ye Ma, Sheana Budhoo

**Affiliations:** 1 Medicine and Surgery, Wyckoff Heights Medical Center, Brooklyn, USA; 2 Internal Medicine, Wyckoff Heights Medical Center, New York, USA; 3 Medical Student/Internal Medicine, Touro College of Osteopathic Medicine, Harlem, USA

**Keywords:** necrotizing fasciitis (nf), perineal infection, scrotal swelling

## Abstract

Fournier’s gangrene (FG) is a rapidly progressive necrotizing infection involving the perineal and genital regions and is associated with significant morbidity and mortality. It is uncommon among adolescents and is typically linked to underlying comorbidities such as diabetes mellitus, immunosuppression, or trauma. We report a case of FG in a 19-year-old male with poorly controlled diabetes who required multiple surgical debridements. Wound cultures identified *Actinomyces europaeus*, an emerging but uncommon pathogen in necrotizing soft-tissue infections. Early initiation of broad-spectrum antimicrobial therapy combined with prompt surgical intervention was critical to disease control in this patient. This case underscores the need for heightened clinical suspicion in young patients with metabolic risk factors and highlights the importance of multidisciplinary management and strict glycemic control to improve clinical outcomes.

## Introduction

Fournier’s gangrene (FG) is a rapidly progressive, life-threatening form of necrotizing soft-tissue infection involving the perineum, genitalia, and perianal region. It represents a subtype of necrotizing fasciitis characterized by polymicrobial infection, tissue ischemia, and widespread fascial necrosis driven by synergistic aerobic and anaerobic organisms. Bacterial toxins and inflammatory mediators lead to microvascular thrombosis, impaired tissue perfusion, and rapid spread along fascial planes, often out of proportion to early cutaneous findings. Despite advances in critical care, antimicrobial therapy, and surgical techniques, reported mortality rates remain high, ranging from 20% to 40% [1].

Although Fournier’s gangrene classically occurs in older men, its occurrence in younger patients remains uncommon and is usually associated with significant underlying risk factors. Diabetes mellitus is the most frequently associated comorbidity, contributing through impaired neutrophil function, microvascular disease, and a favorable environment for anaerobic bacterial growth [2]. Early diagnosis is challenging due to nonspecific initial symptoms, and delays in surgical debridement are strongly associated with worse outcomes.

*Actinomyces europaeus*, first described in 1997, is an anaerobic Gram-positive bacillus traditionally linked to breast abscesses and localized soft-tissue infections. More recently, it has been implicated in invasive infections, including necrotizing fasciitis and rare cases of FG [3]. *Actinomyces europaeus* is a rare cause of necrotizing soft-tissue infections, with only a limited number of cases reported in the literature. Its isolation in this case highlights the expanding microbiologic spectrum of FG and supports emerging evidence that A*. europaeus* may function as a true pathogen rather than a colonizer in severe anaerobic soft-tissue infections.

## Case presentation

A 19-year-old male with a history of diabetes mellitus presented on June 26, 2025, with a 5-day history of worsening scrotal swelling and pain. He had been evaluated in the emergency department (ED) two days earlier but left against medical advice to attend school. Although he reported taking the prescribed trimethoprim-sulfamethoxazole, his symptoms progressed. He denied fever, chills, urinary symptoms, discharge, trauma, or prior similar episodes.

On examination, he appeared visibly distressed from severe scrotal pain. The penis was uncircumcised. The left testis was nonpalpable due to marked swelling; the right testis was palpable and non-tender. The scrotum was diffusely enlarged, erythematous, and exquisitely tender, more pronounced on the left, with fluctuance along the inferior aspect. The perineum was nontender. The left inguinal region was erythematous with palpable lymphadenopathy. These findings raised strong concern for FG, and Urology was urgently consulted.

Broad-spectrum intravenous antibiotics were initiated, and the patient underwent emergent surgical debridement on June 27, 2025, followed by a second-look procedure on June 30, 2025. Intraoperative cultures were processed via Matrix-Assisted Laser Desorption/Ionization-Time of Flight (MALDI-TOF) mass spectrometry, which identified a heavy growth of Gram-positive bacilli, specifically, *Actinomyces europaeus*, alongside rare Staphylococcus epidermidis.

Laboratory studies revealed a glycated hemoglobin (HbA1c) of 12.3% and a C-peptide of 0.60 ng/mL, indicating poorly controlled diabetes with reduced endogenous insulin secretion. He was started on basal-bolus insulin, and Endocrinology optimized glycemic management. The Fournier’s Gangrene Severity Index (FSGI) score was 5, which reflected a favorable prognosis.

**Figure 1:**
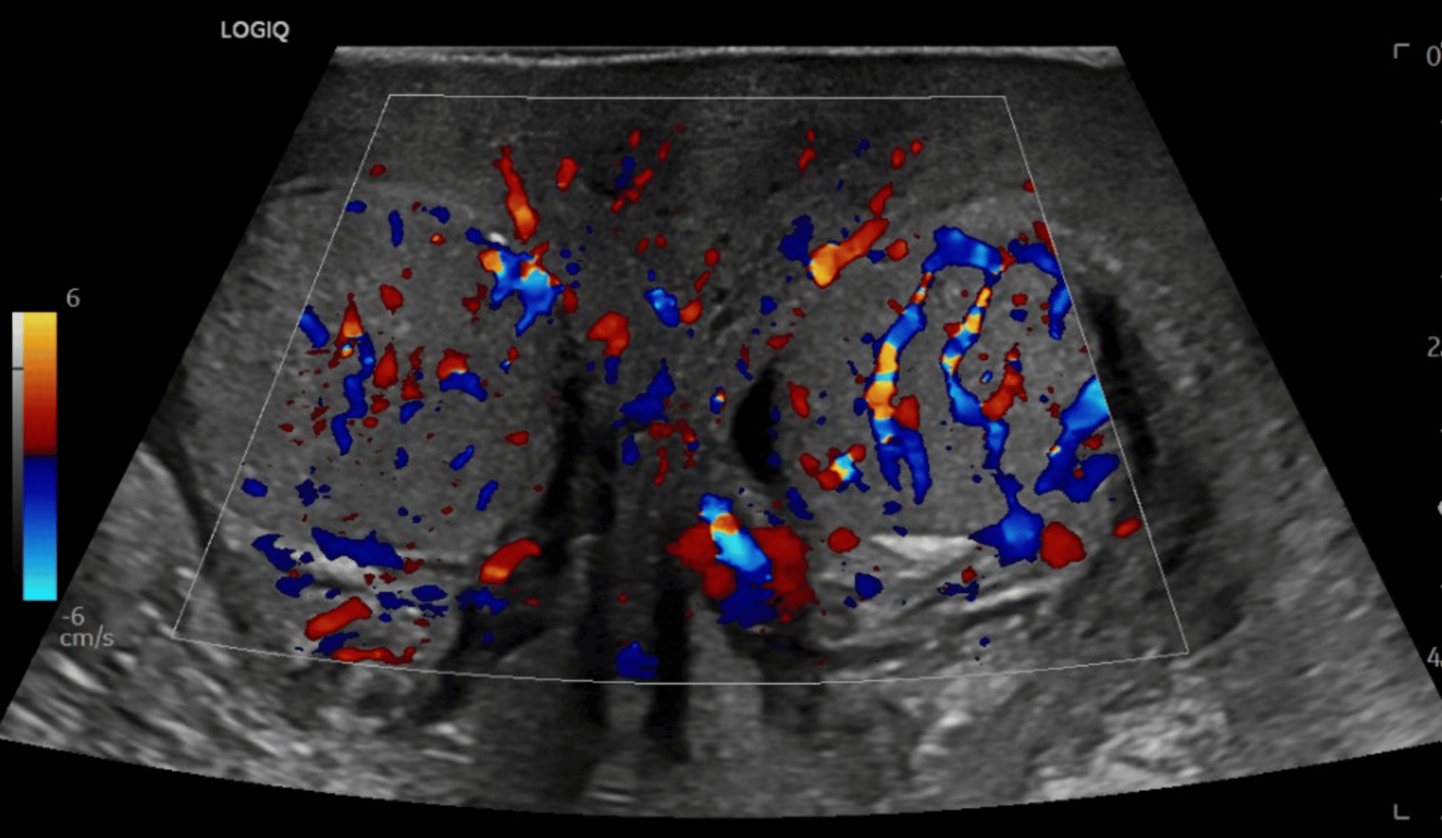
Ultrasonogram of scrotum: slightly hyperemic left testicle without focal lesion, which could be secondary to the scrotal swelling vs orchitis.

**Figure 2:**
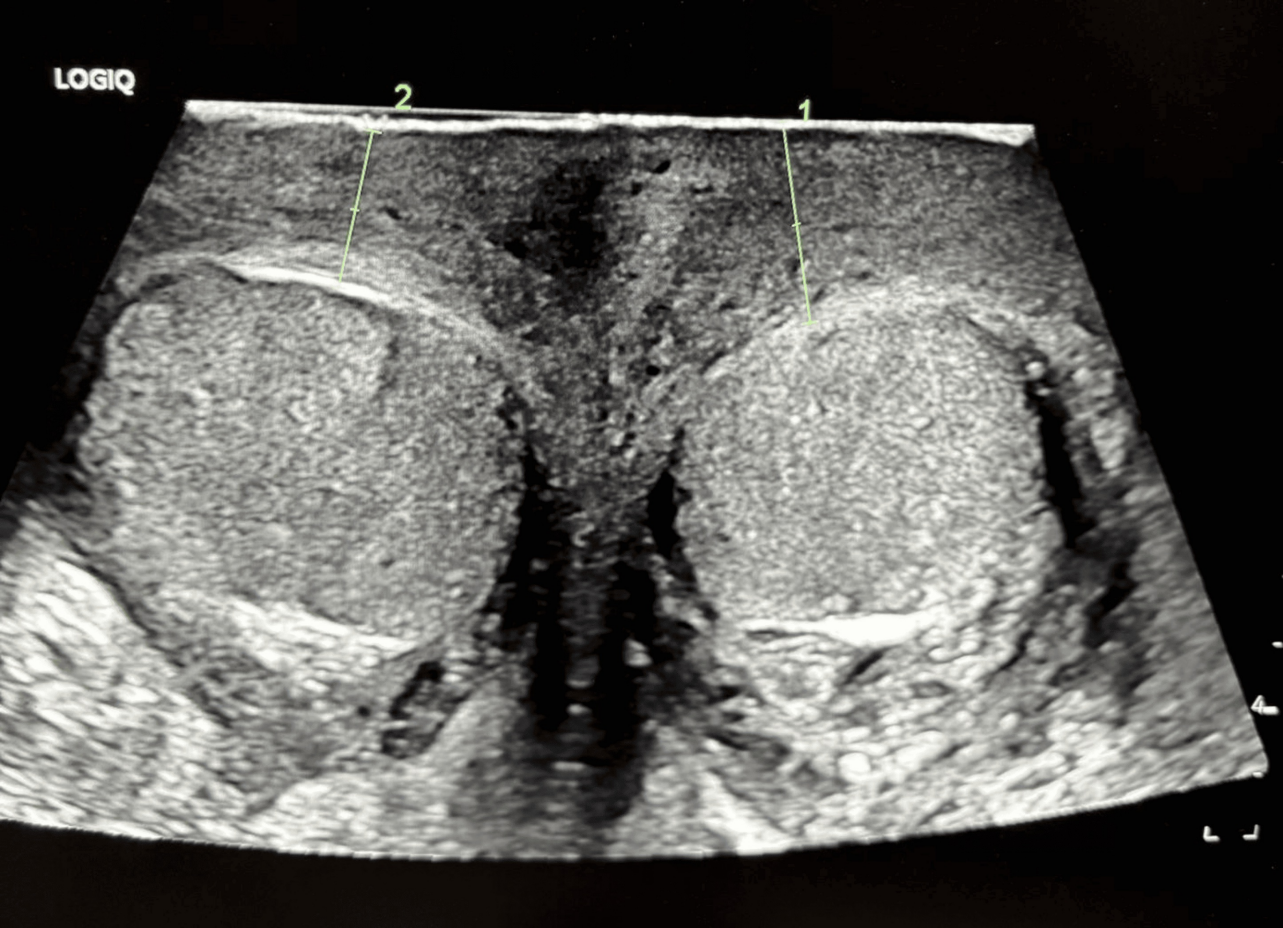
Ultrasonogram of scrotum: diffuse scrotal swelling without cutaneous abscess (area within 1 and 2).

Imaging with scrotal ultrasonography showed diffuse scrotal swelling with slightly hyperemic left testicle without a focal lesion (Figures 1, 2).

**Figure 3:**
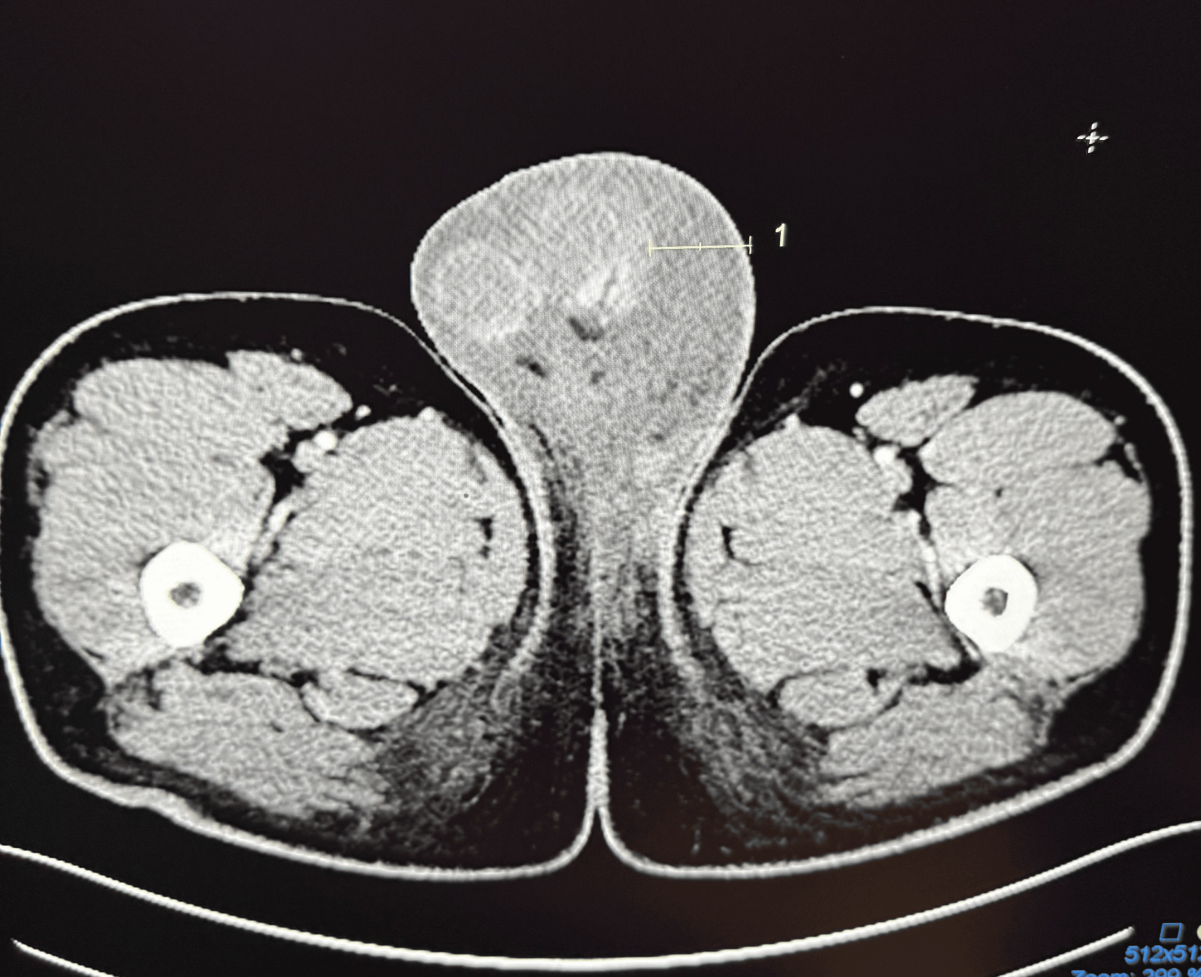
Computed tomography of abdomen and pelvis with contrast: pronounced scrotal swelling, left greater than right, with inflammatory changes (area within number 1) tracking into groins; no drainable abscess in the image area.

**Figure 4:**
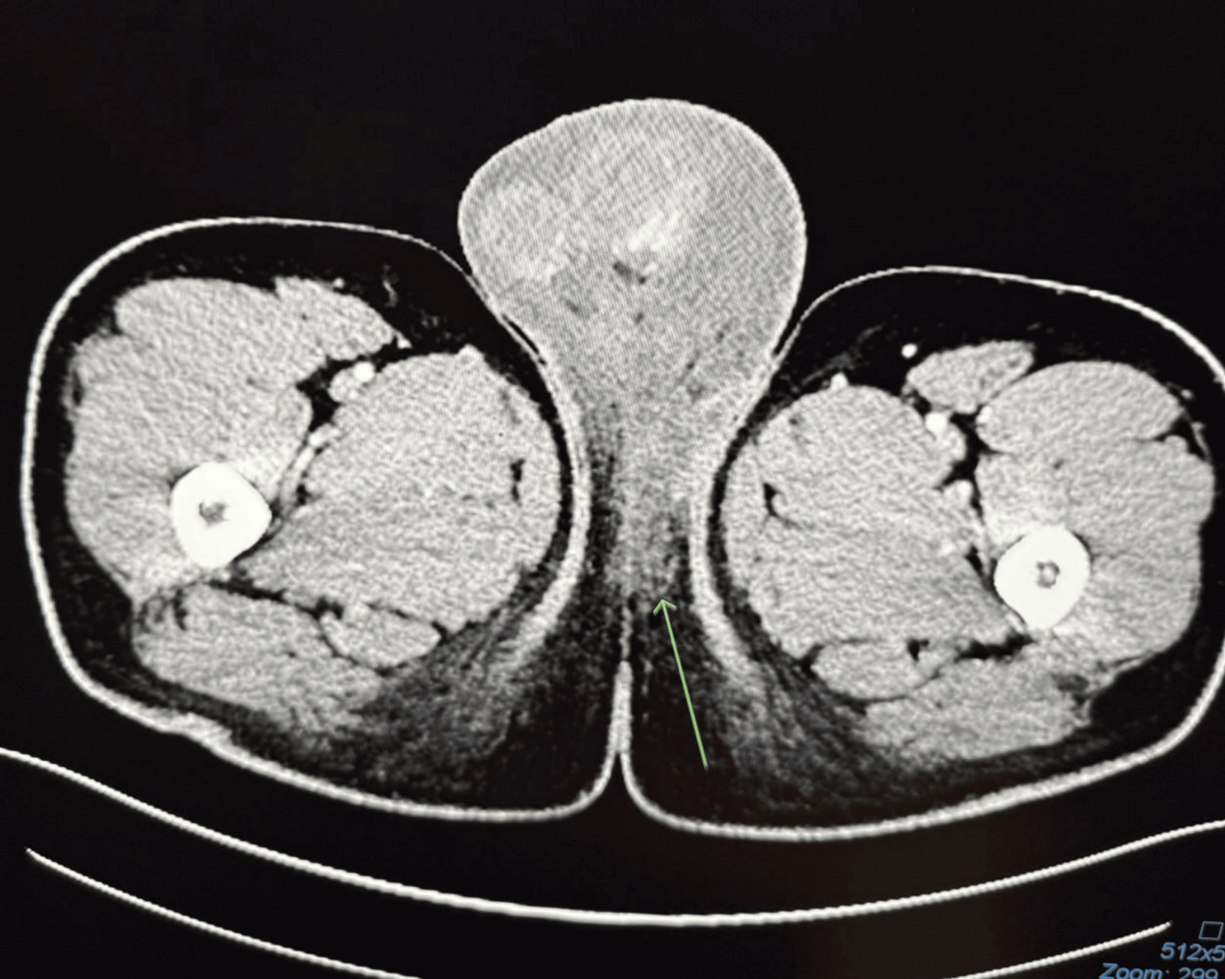
Computed tomography of abdomen and pelvis with contrast: pronounced scrotal swelling, left greater than right, with inflammatory changes tracking into groins (green arrow); no drainable abscess in the image area.

Computed tomography of the abdomen and pelvis with contrast demonstrated extensive scrotal edema with inflammatory involvement of both inguinal regions but no abscess or torsion (Figures 3, 4).

**Figure 5:**
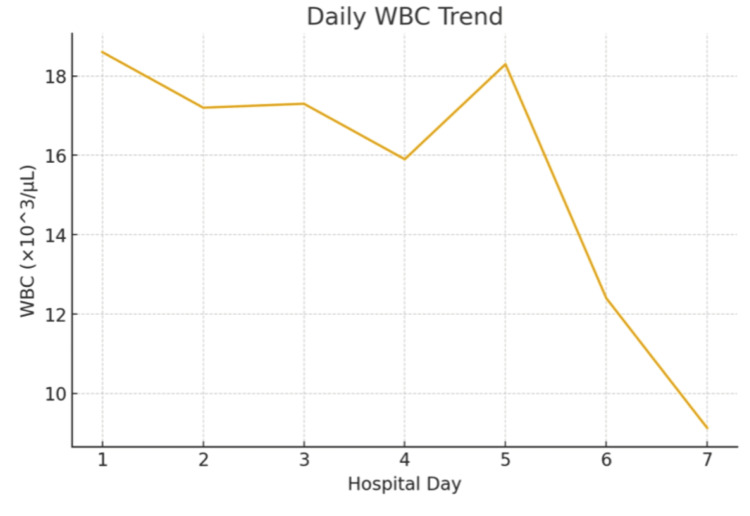
WBC trend during hospital stay.

**Figure 6:**
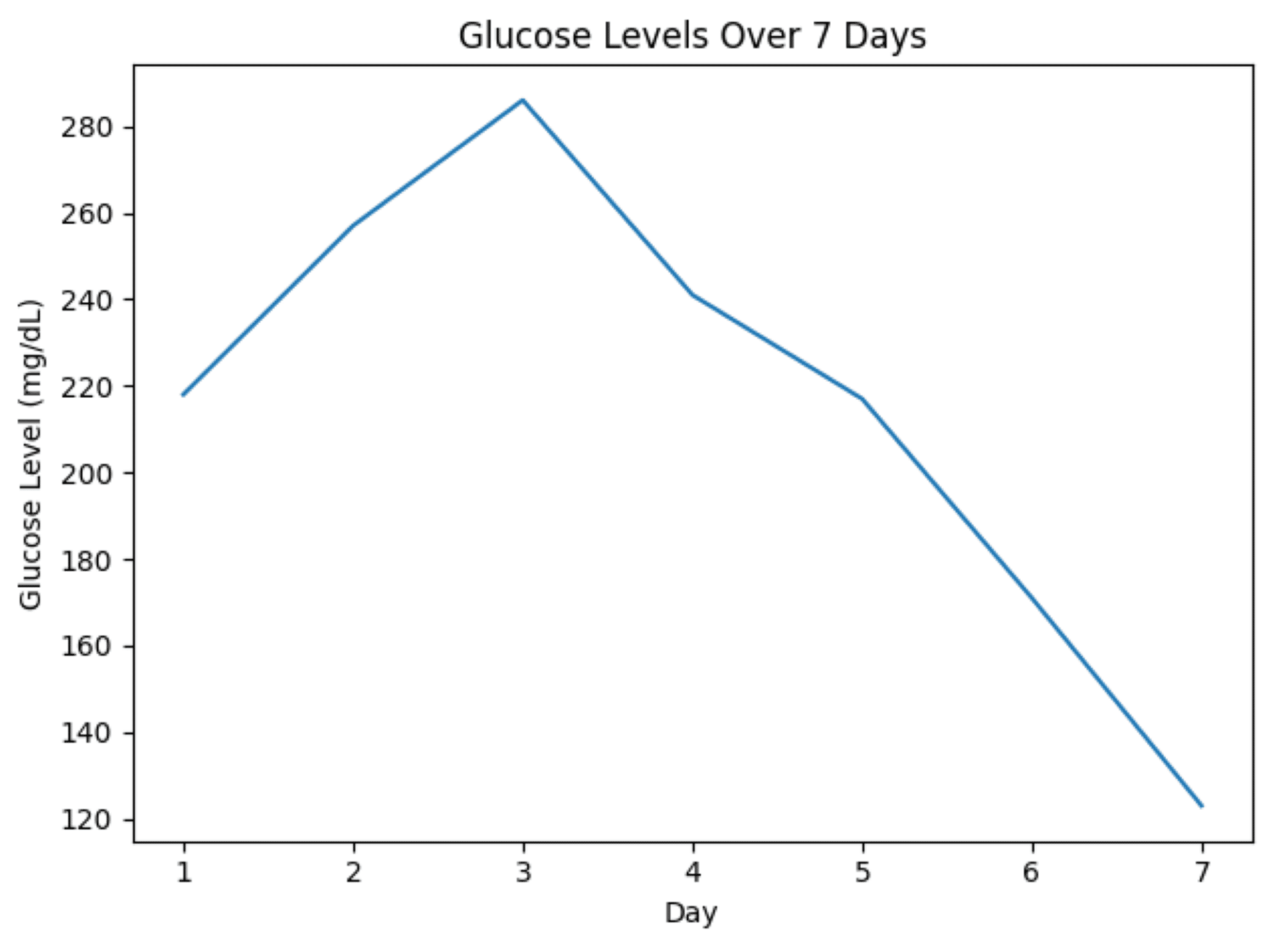
Glucose trend during hospital stay.

The patient remained hemodynamically stable throughout hospitalization. His wound improved following serial debridements. His WBC (Figure 5) and blood glucose (Figure 6) were gradually going down. The Infectious Disease team recommended a four-week course of oral amoxicillin-clavulanate at discharge.

Before discharge, he received detailed wound-care training. He was discharged on oral antibiotics with home health support and close follow-up with Urology, Infectious Disease, Endocrinology, and primary care.

Over the subsequent four months, the wound healed completely, and his diabetes improved substantially with consistent insulin use and endocrinology clinic follow-up. No recurrence of infection occurred.

## Discussion

Fournier’s gangrene is a rapidly progressive, polymicrobial necrotizing fasciitis of the perineal region associated with high morbidity and mortality. Synergistic aerobic and anaerobic organisms produce tissue-degrading enzymes and toxins that induce microvascular thrombosis and ischemia, facilitating rapid spread along fascial planes and leading to extensive tissue necrosis and systemic toxicity [1,2,4]. *Actinomyces europaeus *is increasingly recognized as a pathogen capable of causing severe soft-tissue infections, including FG [3]. Although uncommon, its presence reinforces the need for broad anaerobic coverage and culture-directed therapy. Diabetes mellitus is one of the most significant predisposing factors due to impaired immunity, vascular insufficiency, and delayed wound healing [2,5]. Immunosuppression, trauma, and delayed presentation further increase the risk [6].

Fournier’s gangrene typically presents with severe perineal or genital pain, swelling, and erythema, often with pain out of proportion to physical findings. Because necrosis and crepitus are late findings, their absence can frequently underestimate the true extent of the deep facial involvement [2, 7, 8]. The clinical hallmark of FG is severe pain out of proportion to physical findings, followed by swelling, erythema, and rapid tissue destruction. The diagnosis of Fournier’s gangrene is challenging due to its early nonspecific presentation, occurrence in atypical patient populations, limited sensitivity of laboratory and imaging studies, and the tendency for superficial findings to underestimate the extent of deep fascial involvement [2]. Delay in diagnosis substantially increases the risk of multiorgan involvement and death.

Management of FG requires prompt recognition and immediate intervention. Early and aggressive surgical debridement remains the cornerstone of management, often requiring multiple procedures [7]. Reported cases in the literature remain limited; however, available reports describe aggressive local tissue invasion that requires repeated surgical debridement and prolonged antibiotic therapy [8]. Broad-spectrum empiric antimicrobial therapy covering Gram-positive, Gram-negative, and anaerobic organisms should be initiated promptly and later tailored based on culture results [4]. Infections involving *Actinomyces* species may require prolonged, culture-directed antibiotic therapy [8]. Supportive care, including hemodynamic stabilization, intensive glycemic control, and close monitoring in a critical care setting, plays a crucial role in improving outcomes.

Mortality rates range from 20% to 40% depending on comorbidities, extent of infection, and time to intervention [9, 10]. Prognostic factors associated with increased mortality include advanced age, renal impairment, septic shock, coagulopathy, and low hematocrit [11]. 

The Fournier’s Gangrene Mortality Index (FGMI) score serves as a reliable predictor of mortality and can be utilized during the initial clinical assessment of patients with FG. The FGMI, developed in a 2024 study in *Diagnostics*, demonstrated that albumin and creatinine are key predictors of mortality, with hypoalbuminemia serving as a significant independent predictor [12]. Broader *Actinomyces *species are known to cause chronic suppurative infections [13].

This case adds to the limited literature on FG caused by *Actinomyces europaeus *and highlights several important clinical considerations: FG can occur in adolescents with poorly controlled diabetes; *Actinomyces europaeus *may serve as a primary pathogen; and rapid surgical and medical management is essential to prevent mortality.

## Conclusions

Fournier’s gangrene (FG) can occur in adolescents with poorly controlled diabetes mellitus and should be considered in young patients presenting with rapidly progressive perineal infections. *Actinomyces europaeus *is an emerging and underrecognized pathogen associated with necrotizing soft tissue infections, including FG. Early diagnosis and prompt, aggressive surgical debridement remain essential for survival. Multidisciplinary management, including infectious disease consultation and strict glycemic control, significantly improves clinical outcomes. Increased awareness of atypical pathogens and presentations may facilitate earlier intervention and reduce morbidity and mortality in this high-risk population.
